# Ice mass loss sensitivity to the Antarctic ice sheet basal thermal state

**DOI:** 10.1038/s41467-022-32632-2

**Published:** 2022-09-14

**Authors:** Eliza J. Dawson, Dustin M. Schroeder, Winnie Chu, Elisa Mantelli, Hélène Seroussi

**Affiliations:** 1 Department of Geophysics, Stanford University, Stanford, CA USA; 2 Department of Electrical Engineering, Stanford University, Stanford, CA USA; 3 School of Earth and Atmospheric Science, Georgia Institute of Technology, Atlanta, GA USA; 4 Institute for Marine and Antarctic Studies, University of Tasmania, Hobart, TAS Australia; 5 The Australian Centre for Excellence in Antarctic Science, University of Tasmania, Hobart, TAS Australia; 6 Thayer School of Engineering, Dartmouth College, Hanover, NH USA

**Keywords:** Cryospheric science, Climate and Earth system modelling

## Abstract

Sea-level rise projections rely on accurate predictions of ice mass loss from Antarctica. Climate change promotes greater mass loss by destabilizing ice shelves and accelerating the discharge of upstream grounded ice. Mass loss is further exacerbated by mechanisms such as the Marine Ice Sheet Instability and the Marine Ice Cliff Instability. However, the effect of basal thermal state changes of grounded ice remains largely unexplored. Here, we use numerical ice sheet modeling to investigate how warmer basal temperatures could affect the Antarctic ice sheet mass balance. We find increased mass loss in response to idealized basal thawing experiments run over 100 years. Most notably, frozen-bed patches could be tenuously sustaining the current ice configuration in parts of George V, Adélie, Enderby, and Kemp Land regions of East Antarctica. With less than 5 degrees of basal warming, these frozen patches may begin to thaw, producing new loci of mass loss.

## Abstract (web summary)

This study uses ice sheet modeling experiments to show that thawing portions of the Antarctic ice sheet bed can increase century-scale mass loss, particularly in the Wilkes and Enderby Land regions of East Antarctica.

## Introduction

**Fig. 1:**
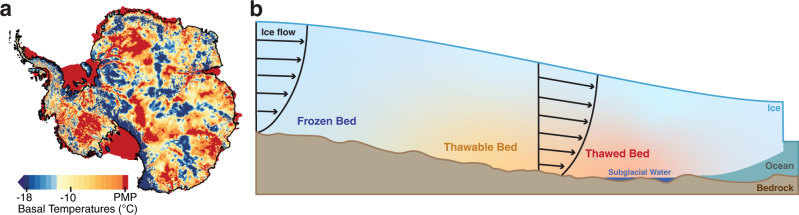
Basal thermal state of Antarctica. **a** Model derived basal temperatures relative to the pressure melting point (PMP) generated from our Ice-sheet and Sea-level System Model (ISSM) initialization. **b** Conceptual diagram of the transition from frozen to thawed bed regions. Thawable bed refers to regions where the basal temperatures are within a few degrees below the pressure melting point. Sketched velocity profiles (arrows) show the onset of basal sliding as the bed thaws.

Across the Antarctic ice sheet, the temperature at the ice-bed interface – basal temperature – is critical in maintaining the ice sheet’s configuration through its influence on basal sliding, subglacial hydrology, and ice-flow regime^1–4^. While frozen bed conditions beneath the continental interior^4–6^ inhibit basal sliding, basal temperatures at, or close to, the pressure melting point (PMP) enable the fast-flow of glaciers and ice streams near the ice-sheet margin^1,7^. In some regions, thawed-bed outlet glaciers or ice streams are interleaved by frozen-bed ice rises or ice ridges^8,9^, resulting in frozen and thawed basal regimes in close proximity to each other (Fig. 1a).

It is possible that Antarctica’s basal thermal state could evolve on centennial timescales. Considering the continuous increase in atmospheric^10,11^ and oceanic warming^12^, the present ice sheet extent may be lagging in its response to external forcing^13^. External atmospheric and oceanic forcing can drive thinning and recession of ice shelves, reduce buttressing, and accelerate upstream ice^14–17^, which could alter the grounded ice basal thermal state as it adjusts to these environmental changes. Additionally, internal ice thermo-frictional feedbacks could create instabilities causing frozen-bed regions within a few degrees below the PMP to spontaneously thaw^18–21^. In these subfreezing regions (i.e., frozen areas with basal temperatures near the PMP), self-reinforcing feedbacks^22^ mediated by temperature-dependent sliding^23^ could lead to the spontaneous onset of wet-bedded, fast flowing ice and the emergence of ice streams^21^. However, the effect of changes in the basal thermal state has not been constrained by observations or modeling^24,25^, so the impact on the dynamics and stability of the Antarctic Ice Sheet remains unknown.

**Fig. 2:**
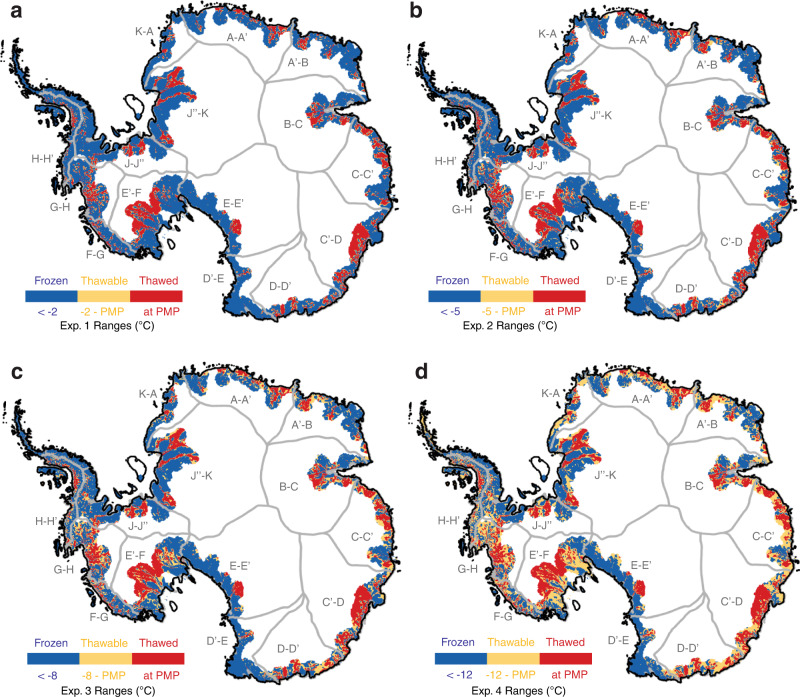
Basal temperature ranges used to configure the thaw experiments. **a** Map of experiment zone (colored periphery region) divided into frozen, thawed, and thawable bed regimes for Exp. 1. The frozen, thawable, and thawed bed ranges come from our ISSM derived basal temperatures. The experiment zone is defined as the portion of each Antarctic basin that lies within 100 km of fast flowing ice (>100 m/yr surface velocity). The gray outlines show the ice drainage basin boundaries and labels are the basin IDs (A-K) produced by E. Rignot and J. Mouginot and used by IMBIE-3^33^. **b–d**) Same as **a** but for exp. 2, Exp. 3, and Exp. 4.

Temporal changes in the basal thermal state can involve two processes: basal thawing and refreezing. While both processes can affect the behavior and evolution of ice sheets, here we focus on whether basal thawing could impact the century-scale evolution of the ice sheet mass balance. To distinguish the parts of the bed that could be vulnerable to thawing, we introduce the term *thawable* to refer to regions where the bed is frozen but within a few degrees below the PMP (Fig. 1b). To identify thawable-bed regions that could transition to thawed-bed, we focus on areas of Antarctica that are within 100 km of fast flowing ice (>100 m/yr surface velocity), where recent mass changes have been observed due to external climate forcing^16^. These areas form the *experiment zone* shown as the colored periphery region in Fig. 2a–d.

In this study, we set up four model experiments with different thawable amplitudes ranging from −2 °C – PMP to −12 °C – PMP to examine the ice sheet sensitivity to various levels of basal thawing in the experiment zone (Table 1 and Fig. 2). We use the state-of-the-art Ice-sheet and Sea-level System Model (ISSM), an open-source finite-element ice sheet model, that simulates the 3D thermomechanics of ice flow^26^. We first use ISSM to calculate the present-day steady state ice sheet basal temperatures (Fig. 1a), and then we use this output to initialize the subsequent 100-year thaw experiments (see Methods).

**Table 1:** Basal temperature ranges defined for each thaw experiment

Thaw experiment	Frozen range	Thawable range	Thawed range
Exp. 1	<−2 °C	−2 °C — PMP	At PMP
Exp. 2	<−5 °C	−5 °C — PMP	At PMP
Exp. 3	<−8 °C	−8 °C — PMP	At PMP
Exp. 4	<−12 °C	−12 °C — PMP	At PMP

*PMP* pressure melting point.

## Results

### Experiment design to simulate basal thawing

We use a novel approach to simulate basal thawing in thawable-bed regions. A frozen-to-thawed bed transition would, in theory, reduce basal friction and accelerate sliding^27^. However, most large-scale ice sheet models lack subfreezing sliding physics, and even those that include it^28^ lack the full treatment of ice thermomechanics required to capture the temporal evolution of these subfreezing regions^22^. Thus, we develop a method (described below) to reduce the modeled basal friction coefficient to capture the effect of a frozen-to-thawed transition in enhancing the slipperiness at the bed. This method enables us to conduct a first-of-its-kind investigation of the effect of basal thawing on ice mass loss, even though it lacks thermo-frictional feedback mechanisms that are needed to fully resolve the ice sheet response to basal thaw.

We run four model experiments with the following thawable amplitudes within the experiment zones: For Exp. 1, we define the thawable-bed regions as the regions where basal ice temperature amplitude is −2 °C – PMP; Exp. 2 is −5 °C – PMP; Exp. 3 is −8 °C – PMP, and Exp. 4 is −12 °C – PMP (Table 1 and Fig. 2). These thawable amplitudes were selected based on the spatial distribution of our present-day ISSM basal temperatures: −2 °C was the smallest amount of warming needed to thaw regions of the bed adjacent to already thawed areas; −12 °C was the amount of warming needed to thaw parts of frozen-bedded ice rises and ice ridges in the experiment zones. Including a range of experiments also accounts for uncertainties in the modeled basal temperatures and allows us to investigate interesting regions where the basal temperature could in reality be warmer or colder than our model implies. The range of thawable amplitudes is also compatible with previous experimental constraints on the range of temperatures below the melting point where subfreezing sliding is expected^29,30^.

**Fig. 3:**
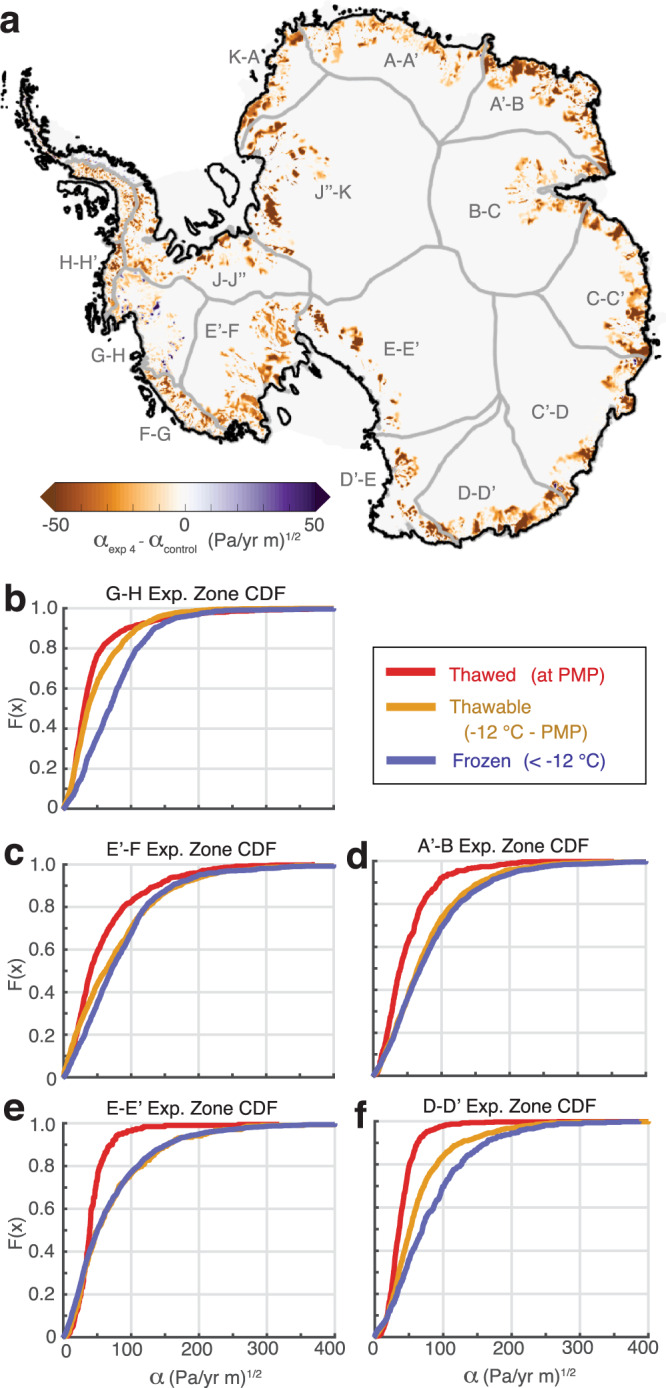
Basal friction coefficient distributions. **a** Prescribed change in basal friction coefficients for Exp. 4 (${\alpha }_{{{\exp }}4}-\,{\alpha }_{{{{{{\rm{control}}}}}}}$) in thawable-bed regions of the experiment zone. **b–f** Plots of the empirical cumulative density function (CDF) for *α* in the thawed, thawable, and frozen regions for a subset of drainage basins (see Supplementary Fig. 5 for all drainage basins). The CDFs plotted here are for Exp. 4; see Supplementary Fig. 6 for a comparison to the other three experiments.

To prescribe thawing in the thawable regions, we first define basal sliding in the model set-up using a Weertman-type friction law^31^, which is implemented in ISSM^26^ as

1 $${\tau }_{b}={-\alpha }^{2}{{{{{{\boldsymbol{v}}}}}}}_{{{{{{\boldsymbol{b}}}}}}}$$

where ${\tau }_{b}$ is the basal shear stress (kPa), ${{{{{{\boldsymbol{v}}}}}}}_{{{{{{\boldsymbol{b}}}}}}}$ is the basal ice velocity vector (m/yr), and *α* is the friction coefficient ((Pa/yr m)^1/2^). We then extract the model inferred *α* values^32^ in the experiment zone and group them into thawed, thawable, and frozen ranges based on the basal temperature output from the model initialization. For each temperature range, we present the corresponding *α* values as empirical Cumulative Density Functions (CDFs) (Fig. 3 for exp. 4, also see Supplementary Fig. 6 for comparison to exp 1–3). In general, across all of the Antarctic drainage basins (defined by E. Rignot and J. Mouginot and used by IMBIE-3^33^), there are higher *α* values in frozen and thawable regions and lower *α* values in thawed regions (Fig. 3). This pattern in the CDFs is an expression of the different subglacial environments reflected in the friction coefficient values, including the influences of the underlying geology. Using this empirical *α*-to-thermal-regime relationship, we can simulate the effect of basal thaw on the rate of basal sliding by altering *α* values to transition thawable-bed regions to being thawed (Fig. 3a). In each of the four thaw experiments, we prescribe thawing by replacing the thawable *α* values in experiment zones with the thawed *α* values that correspond to the same *F(x)* in the CDF (Fig. 3b–f). We refer to this modified *α* in the thawable regions as ${\alpha }_{{{\exp}}1-4}$. The ${\alpha }_{{{\exp }}1-4}$ are prescribed as a step change from our initial configuration (see Methods), and then sustained for 100 years in transient, forward-type simulations. A control simulation (i.e., with no change in *α*) is run for 100 years to provide a benchmark to the other experiments. The ice sheet geometry, velocity, and grounding lines are allowed to evolve in time in all simulations.

To examine the robustness of the experimental set-up, we perform three sets of additional experiments: (i) replacing Eq. (1) with a Budd^34^ friction law to capture drainage-related effects, (ii) modification of *α* by a prescribed percentage of its original value (rather than the CDF method), and (iii) extending thaw areas further into the ice sheet interior. These additional experiments are discussed in Supplementary Note 1 and summarized in Supplementary Table 1.

### Effect of basal thaw on 100-year mass loss trends

**Fig. 4:**
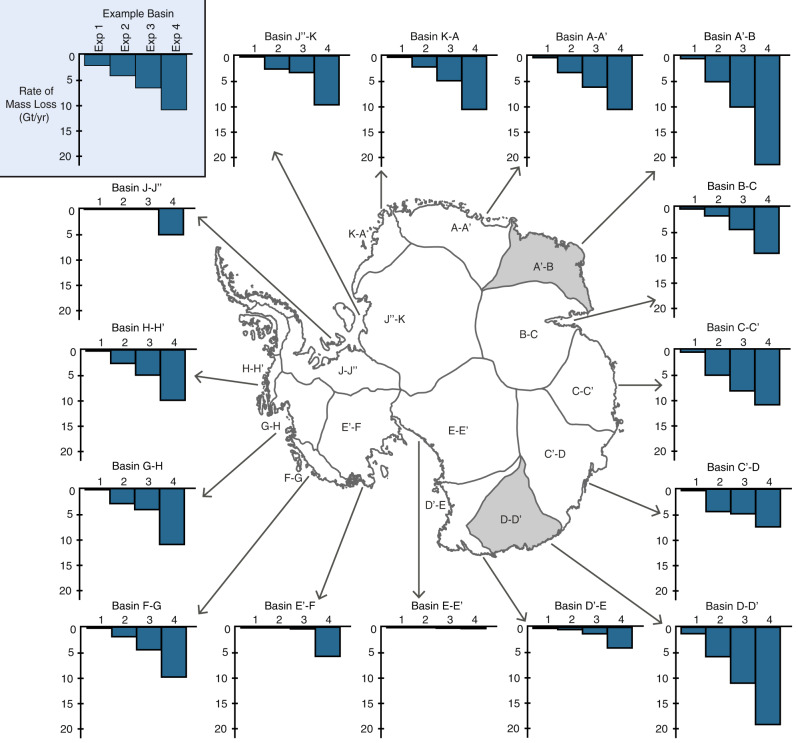
Mass loss resulting from 100-year thawing experiments. The bar charts show the change in mass above flotation relative to the control run for the four thaw experiments in each drainage basin. The center map highlights the two drainage basins with the largest mass loss (shaded gray).

Overall, our thaw experiment results show a greater loss in total mass above flotation compared to the control simulation where *α* values are held constant. On a continental scale, more aggressive thawing produces more mass loss relative to the control for all basins (Fig. 4). At the basin scale, there are notable regional differences in the amplitude and spatial pattern of the mass loss response per thaw experiment (Fig. 4). For additional context, we also plot the changes in ice thickness that result from the experiments in Supplementary Fig. 7.

In West Antarctica, the mass loss in the Siple Coast and Ellsworth Mountain basins (E’-F and J-J” in Fig. 4) highlight the regional sensitivity of ice rises to the basal thermal conditions. While both basins have almost no mass loss for Exp. 1 through Exp. 3, Exp. 4 shows a jump in the rate of mass loss up to 5 Gt/yr. This jump coincides with a larger extent of thawing that includes parts of the Bungenstock, Siple Dome, Conway, and Engelhardt ice rises where previous ice-penetrating radar sounding observations suggest a presently frozen bed^1,8,9,35,36^. In contrast, in the Amundsen Sea Embayment (G-H), Marie Byrd Land (F-G), and the Bellingshausen Sea (H-H’) regions, our modeling results display little to intermediate mass loss (0.5–2.5 Gt/yr) with moderate thawable areas in Exp. 1 and Exp. 2, and up to about 4 to 5 times that for the most extreme thawing scenario represented by Exp. 4. Perhaps surprisingly, in the Amundsen Sea Embayment where recent observations already show significant mass loss (>100Gt/yr)^33^, our results suggest that this region is relatively insensitive to further basal thawing.

In East Antarctica, along the Wilkes Land coast up to MacRobertson Land and the Dronning Maud through Shackleton-Pensacola regions (C’-D, C-C’, B-C, A-A’, K-A, and J”-K in Fig. 4) our results suggest similar, moderate rates of mass loss (up to ~10 Gt/yr) in response to thawing (Fig. 4). Along the Wilkes Land coast, the rate of loss is comparable to recent observations^16,33^. MacRobertson Land and Dronning Maud through Shackleton-Pensacola regions show higher rates of mass loss and sensitivity to thawing, since recent observations show that the regions are roughly near balance^33^. In contrast, our model shows the Transantarctic mountains (E-E’ and D’-E) have the lowest relative mass loss, which is somewhat expected since the basal temperatures are well below −12 °C. Notably, our model highlights that the Enderby-Kemp and George V - Adélie Land regions (D-D’ and A’-B) of East Antarctica would likely undergo the most mass loss and be most sensitive to changes in basal thawing. These regions lose ~20 Gt/yr for Exp. 4, doubling the rate of other nearby basins. This number is also an order of magnitude higher than the observed dynamic loss for these regions over the last four decades^33^.

In addition to the main experiments described above, we perform an experiment similar to Exp. 4 but using a Budd friction law^34^ to examine the sensitivity of our model results to the choice of friction law (Supplementary Note 1(i)). These sensitivity tests show similar rates of total mass loss to the Weertman sliding simulation overall with some regional differences in mass loss in the Bellinghausen Sea, Marie Byrd Land, Donning Maud Land, George V Land, and Wilkes Land regions (H-H’, F-G, A-A’, D-D’, and C’-D shown in Supplementary Fig. 1). Previous work has found that the choice of friction law has a large effect on centennial mass loss projections^37,38^. Even with this consideration, our projections predict high rates of mass loss for George V- Adélie Land and Enderby - Kemp Land for both friction laws, and the addition of Wilkes Land for Budd. This result suggests that East Antarctica’s mass loss response to basal thaw is a robust finding, regardless of the friction law applied.

### Factors influencing the impact of basal thawing

**Fig. 5:**
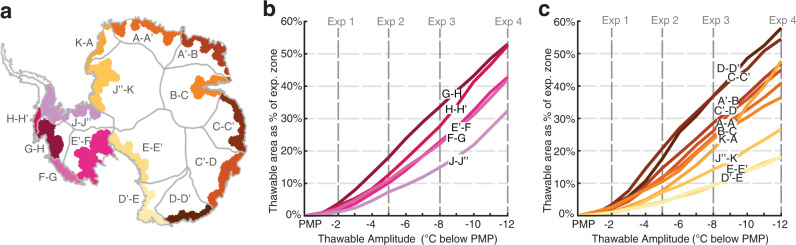
Percentage of thawable area within each drainage basin experiment zone. **a** Map of each basin experiment zone with darker shaded regions indicating basins with a larger spatial extent of thawable area. West and East Antarctic basins are marked with different color scales (pink and orange, respectively). Percentage of total experiment zone area that is in the thawable-bed temperature amplitude (West Antarctica in **b**, East Antarctica in **c**). The vertical dashed lines indicate the amplitudes of the four thaw experiments.

We suggest that the regional variability in mass loss sensitivity to basal thawing from our thawing experiments is due to the interplay of two basin-specific conditions: the shape of the thawable-bed *α* CDF (Fig. 3) and the extent of thawable-bed area in the experiment zone (Fig. 5). A good demonstration of this interplay is the George V - Adélie Land region (D-D’) of East Antarctica. This region has the largest thawable area of any basin (up to ~60% of the total basin area as seen in Fig. 5c) and a relatively large reduction in *α* going from the initial inferred thawable *α* to the thawing experiments ${\alpha }_{{{\exp }}1-4}$ (Fig. 3a, f). These combined conditions drive extensive mass loss. This is concerning because the vast Wilkes Subglacial Basin is located in this region and drained via the Cook and Ninnis glaciers along the George V Land coast^39^. The geometry is such that greater ocean access to the ice sheet may have enabled the basin to undergo substantial retreat in the past^40,41^, and recent acceleration in this region also suggests a risk of future destabilization^42^. Furthermore, small volumes of ice along the coast near the Cook and Ninnis glaciers – termed *ice plugs*^39^ – could potentially destabilize the catchment leading to self-sustained discharge of the basin if removed. Our results suggest that localized frozen-bed patches may have an important role in holding ice plugs in place, and thereby help to sustain the current configuration and tenuous stability of the subglacial basin. If these frozen-bed patches undergo thawing, then increased basal sliding could potentially trigger catchment-scale retreat through the removal of critical ice plugs.

Similarly, the Enderby – Kemp Land basin (A’-B) has substantial thawable areas (up to ~45% as seen in Fig. 5c) and a large reduction in *α* when transitioning from thawable to thawed, making it particularly vulnerable to mass loss. Subsurface observations, for example provided by ice-penetrating radar sounding, are comparatively sparse in this region, so it remains to be verified how much of its ice-bed interface is presently close to thawable. While mass balance observations in Enderby Land show a small amount of mass gain over the last decade^33^, there is past evidence of glacier retreat in this region in the 1970s and 1980s^33,42,43^. A recent study at Shirase glacier – the fastest outlet glacier in the Enderby Land basin – found strong ice-driven melting beneath the glacier tongue^44^. This provides evidence that parts of the Enderby Land region could be affected by the inflow of warm ocean water, which plays a key role in ice mass balance.

The relatively low sensitivity to thawing seen in the Amundsen Sea basin (G-H) can be attributed to the basin-specific conditions. While the thawable extent is fairly high (up to ~50% as seen in Fig. 5b), the small mass loss response is due to the similar *α* distributions for thawed and thawable portions of the experiment zone (Fig. 3b). This suggests that the Amundsen Sea basin may have already experienced the majority of its potential thaw-induced changes. Previous observational and modeling studies have shown that much of the bed is already thawed^1,7^, and our findings suggest that additional thawing would likely not have large effects on the mass balance.

## Discussion

Our results show that a warmer basal thermal state could increase mass loss from the ice sheet, which highlights the need to accurately resolve basal temperatures. This need is particularly relevant for George V - Adélie Land (D-D’) and Enderby - Kemp Land (A’-B): these regions comprise ~11% of the total ice sheet area and could be susceptible to mass loss an order of magnitude higher than the observed dynamic loss over the last four decades^33^ as a result of localized basal thaw. We show that the susceptibility is primarily governed by patches of thawable-bed, which are poorly constrained due to the challenging nature of observing the ice-bed interface. Ice boreholes can provide profiles of the ice sheet temperature that constrain the ice-sheet basal thermal state^1^, but there are very few boreholes across the continent. Radar sounding or seismic observations can be used in conjunction with ice sheet modeling to constrain the basal thermal state on a regional scale^35,45,46^, however, the limited coverage of the data has hindered validation and application at a continental scale. Ambiguity regarding the basal thermal conditions is concerning in the context of next-century sea level rise projections since our results suggest that the basal thermal regime can exert a strong control on the expected mass loss. Additional ground truthing to constrain where thawable-bed patches exist across the ice sheet should be a top priority for future field studies.

In addition, our results highlight the need for advances in modeling the processes leading to thawing and the ice sheet response to environmental forcing^24^. Our results likely represent an upper-bound on expected mass loss, since warmer conditions are introduced abruptly and over the entire thawable regions. In reality, we would expect such changes to happen more progressively, and they may occur in only one region. Further, our experiments are built around an idealized representation of basal thawing that does not account for geometric and thermal feedbacks that could result in the bed eventually refreezing.

To further evaluate the ice sheet sensitivity to basal thawing, we believe there are two key questions to investigate: (1) What physical processes can lead to basal thawing? (2) Under what conditions and over what timescales can a thawed bed be sustained? Modeling the physical processes capable of driving basal thawing would require a continent-scale ice sheet model with Stokes mechanics at high spatial and temporal resolution to resolve sliding onset physics^47^. Our work motivates the need for more model development in this area. The latter question requires an investigation of refreezing. One pathway that could lead to refreezing is a feedback between faster sliding speeds driven by thawing and the ice geometry, where increased flow speed would lead to thinning and therefore larger conductive heat loss to the surface. This process entails a rearrangement of the englacial temperature field, thus our 100-year simulations are probably barely long enough to start seeing the impact. Another pathway is that thawing may imply a large enough reduction of basal friction that frictional heating would no longer be large enough to maintain a positive melt rate, despite the faster sliding speeds. This would be instantaneously apparent in our simulations. Our simulations do not show refreezing in thawable areas, which suggests that they could remain thawed after their initial frozen-to-thawed transition (Supplementary Note 4). However, as discussed above, the advances in ice mechanics and sliding onset physics for continent-scale ice sheet simulation codes are needed to examine the self-reinforcing feedbacks that could either lead to the ice thinning and eventually result in refreezing to the bed or permit sustained thawed conditions if frictional heating is great enough^21,22^.

As a first assessment of ice mass loss sensitivity to basal thawing, our work clearly suggests that there is a relationship between basal thawing and mass loss, particularly in parts of East Antarctica. The considerable mass loss that we find in response to basal thawing raises the possibility that physical processes driving basal thaw are part of a feedback that should be considered as a mechanism contributing to sea level rise. To assess this, we propose that follow-on work should investigate the spatial extent and timescales of physical processes that could potentially force and sustain thawing. Additionally, there is a need for observational constraints of the basal thermal state particularly in the George V, Adélie, Enderby and Kemp Land regions of East Antarctica where our model results show that parts of the bed are within a few degrees of thawing. These advances in observationally constraining the basal thermal state and including the physics of basal thaw in model projections of the Antarctic ice sheet’s future evolution are critical steps to understand the effects of basal thaw, a hitherto neglected process in the context of sea level rise projections.

## Methods

### Model set-up

ISSM is an open-source finite-element ice sheet model that simulates the 3D thermomechanics of ice flow^26^. Ice is treated as a viscous incompressible material with strain and temperature-dependent viscosity following Glen’s Law^48^. For the stress balance calculation, we use a 3-D higher-order approximation to the Full Stokes equations^49,50^. The ice sheet surface is stress-free and at the ice-bed interface we apply a power-law version of the friction law^51^ that is implemented in ISSM in terms of the basal shear stress

2 $${{{{{{\boldsymbol{\tau }}}}}}}_{{{{{{\boldsymbol{b}}}}}}}={-}{\alpha }^{2}{N}^{r}{\Vert {{{{{{\boldsymbol{v}}}}}}}_{{{{{{\boldsymbol{b}}}}}}}\Vert }^{s-1}{{{{{{\boldsymbol{v}}}}}}}_{{{{{{\boldsymbol{b}}}}}}}$$

where ${{{{{{\boldsymbol{\tau }}}}}}}_{{{{{{\boldsymbol{b}}}}}}}$ is the basal shear stress, *α* is the basal friction coefficient, *N* is effective pressure, ${{{{{{\boldsymbol{v}}}}}}}_{{{{{{\boldsymbol{b}}}}}}}$ is the basal velocity vector, and $r=q/p$ and $s=1/p$ are friction law exponents^51^. We explore the sensitivity of the model to the choice of friction law, with a comparison between results obtained with Weertman and Budd friction reported below in Supplementary Note 1(i). In all the experiments presented in the main paper, we set *p* = 1 and *q* = 0 in Eq. (2), which reduces to a linear Weertman friction law^31^. In Supplementary Note 1(i), we perform comparison experiments with *p* = 1 and *q* = 1, which is the form of a linear Budd friction law^34^. *N* is the difference between overburden and subglacial water pressure ($N={g}{\rho }_{i}H-{g}{\rho }_{w}b$), where *g* is gravity, ${\rho }_{i}$ and ${\rho }_{w}$ are the respective densities of ice and water, *H* is the ice thickness, and *b* is the ice basal elevation. Note that this definition of N assumes a perfect connection between the subglacial drainage system and the ocean. By assuming this, we neglect variations in basal pore water pressure that act to reduce the effective pressure exerted by the overlying ice.

We solve for the ice temperature using an enthalpy-based formulation of the unsteady advection-diffusion heat transport problem, which allows for both cold and temperate ice to be included in the energy-conserving framework as described in ref. 52 and implemented in ISSM in ref. 53. For the thermal boundary conditions, the air temperature is imposed at the upper surface^26^. The basal boundary condition for the enthalpy field at each basal location is decided according to ref. 52, depending on the cold or temperate conditions at the ice base. Meltwater at the base can thin with refreezing or thicken with melting but it does not flow since, for simplicity, we do not include a hydrology model to reroute it.

Evolution of the ice sheet surface is determined by mass conservation, following the typical formulation for the mass transport equation^26,32^. The glacier thickness is updated at each time step according to mass conservation. To avoid numerical instabilities, the minimum ice thickness allowed is 10 m. We also allow grounding lines to migrate in our simulations. ISSM migrates grounding lines based on the hydrostatic equilibrium at each time step of the transient ice flow solution. For partially floating elements, we use sub-element parameterization to track the position of the grounding line within the element following the sub-element friction interpolation scheme 1 discussed in ref. 54. For the basal melt interpolation, we do not apply melt on partially floating elements. We apply the mean basal melt rate values to elements when the grounding line retreats.

### Datasets

For the Antarctic ice sheet geometry, we use BedMachine Antarctica v 1 (https://sites.uci.edu/morlighem/bedmachine-antarctica/)^55^. We use surface velocity from the MEaSUREs InSAR-based Antarctic Version 2 velocity map (https://nsidc.org/data/nsidc-0484)^56,57^. The remaining surface conditions are surface temperature and surface mass balance from the 1979–2014 climatological annual mean from MAR v 3.6.4^58^. We use ice shelf basal melt rate values from ref. 59. For our geothermal heat flux, we use the map by ref. 42.

### Model initialization

Our model domain covers the entire Antarctic ice sheet. We use an adaptive mesh with horizontal resolution ranging from 1 km along the coast to capture narrow fast flowing glaciers to 30 km in the continental interior to maintain reasonable computational cost. In the vertical direction, we use 5 layers with quadratic interpolation functions (P1xP2 finite element type)^60^ and the layer spacing is refined towards the bottom (extrusion exponent of 1.2) to better resolve the rapidly changing temperature structure and vertical shearing in the bottom part of the ice thickness. The 3-D mesh comprises ~800,000 prismatic elements.

The initial model configuration is computed via an iterative initialization procedure that seeks to compute the 3D velocity and temperature fields for a known, present-day ice sheet geometry. Initially, we treat the mechanical and thermal problems separately. First we consider the mechanical problem, where we use the same inverse method presented in ref. 32 to minimize the misfit between observed, ${{{{{{\boldsymbol{v}}}}}}}^{{obs}}=({v}_{x}^{{obs}},{v}_{y}^{{obs}}),$ and modeled, ${{{{{\boldsymbol{v}}}}}}=({v}_{x},{v}_{y}),$ present day surface velocities^32^ to infer the basal friction coefficient, *α*, on the grounded ice sheet, and similarly for depth-average ice rigidity, *B*, over ice shelves^61^. The cost function is

3 $${{{{{\mathcal{J}}}}}}({{{{{\boldsymbol{v}}}}}},\alpha )=	 {\gamma }_{1}\frac{1}{2}\int _{{\Gamma }_{s}}{\left({v}_{x}-{v}_{x}^{obs}\right)}^{2}+{\left({v}_{y}-{v}_{y}^{obs}\right)}^{2}{{{{{{\rm{d}}}}}}\Gamma }_{s} \\ 	+{\gamma }_{2}\frac{1}{2}\int _{{\Gamma }_{s}}{{{{\mathrm{ln}}}}}\,{\left(\frac{\sqrt{{v}_{x}^{2}+{v}_{y}^{2}}+\varepsilon }{\sqrt{{v}_{x}^{ob{s}^{2}}+{v}_{y}^{ob{s}^{2}}}+\varepsilon }\right)}^{2}{{{{{{\rm{d}}}}}}\Gamma }_{s}+{\gamma }_{t}\frac{1}{2}\int _{{\Gamma }_{b}}\nabla \alpha \cdot \nabla \alpha \,d{\Gamma }_{b}$$

where γ_1_ is the coefficient for the term representing the absolute difference in velocity, *γ*_2_ is the coefficient for the log of the misfit, and γ_t_ is the coefficient for the regularization term and *ε* is the minimum velocity used to avoid singularities. We choose are *γ*_1_ = 400, *γ*_2_ = 1, and ${\gamma }_{t}=6{{{{{\rm{e}}}}}}-13$ for the basal friction coefficient inversion, which we calibrate with an L-curve analysis and by selecting the coefficients that result in the minimum difference between observed and modeled surface velocities (Supplementary Fig. 9f). For the ice rigidity inversion, *γ*_1_ = 100, *γ*_2_ = 1 and there is no regularization term. For the first iteration, the ice rigidity temperature dependence is calculated from observed surface temperatures, which allows us to compute an initial guess for the depth-resolved ice sheet velocity before we have a model temperature field.

For the thermal problem, we assume a thermal steady state throughout the initialization^53^. We stabilize the heat advection solver with the anisotropic streamline upwind Petrov–Galerkin (SUPG) method which circumvents the problem of an ice sheet’s high aspect ratio by treating the horizontal and vertical directions separately in the stabilization coefficients^62^. For the first iteration step, we solve a diffusion only problem for the temperature field. We then successively iterate our initialization procedure for the mechanical and thermal problem: the ice velocity field is used to update the temperature field by solving the steady, 3-D advection-diffusion problem for temperature^53^, this updated temperature is then used to update the velocity field via the solution of the inverse mechanical problem described above. To ensure full consistency between the thermal regime and the stress balance regime we run multiple iterations of the mechanical and thermal model until the mean basal temperature varies less than 0.05% between iterations. Also, we apply 20% random noise as perturbation to the friction coefficient values on our final two iterations to ensure that the inversion optimization does not get stuck in a local minimum.

Results of our initialization are shown in Supplementary Fig. 9. Our map of pressure adjusted basal temperatures (panel b) reflects surface velocities (panel a), with the pattern of thawed beds below fast flowing glaciers and frozen beds below slow flowing regions. To benchmark our basal temperatures, we compare the thawable range of basal temperature fields with models used in the ISMIP6 experiments^24^ (Supplementary Fig. 9d). The darker pink colors in d denote more agreement amongst models that the bed is thawable. Broadly speaking, our model picks up the same thawable regions as the ISMIP6 models. However, there are a few regions where our model is colder than most ISMIP6 models such as near some glaciers along the Wilkes Land coast and in the fringes of the Siple Coast and Amundsen Sea regions. That being said, there is also a significant amount of variation in basal thermal states between the models used in the ISMIP6 comparison project^24^. To further ascertain uncertainty in our initialization temperature field, we compare profiles of our modeled temperature to borehole temperature measurements at Lake Vostok, South Pole, Dome C, and WAIS Ice Divide^63–66^. In all cases, our modeled temperature field is within a few degrees of observations (Supplementary Fig. 10). Using MAR v 3.6.4^58^ for surface temperature and Shen geothermal heat flux^42^ achieved the best agreement with borehole observations.

For all 100-year experiments, the climate conditions and ice shelf basal melt rates are held constant, so the only perturbations come from *α* modifications. For the control simulation, *α* is unperturbed from its initial inferred values (otherwise the same setup as the experiments). To update the mesh at each time-step of the 100-year simulations, we use the arbitrary Eulerian–Lagrangian method^67^, where the vertical elements adjust according to the ice surface while the horizontal mesh remains fixed. We choose a fixed bimonthly time step (48 steps per year). We chose this fixed time step after testing runs with adaptive time stepping to determine the maximum time step that satisfies numerical stability criteria. The control shows a small increase in the total ice sheet volume above flotation of 0.23% over 100 years (Supplementary Fig. 9e). This small increase in total volume is similar to that found by previous studies: Schlegel et al.^68^ reported a 0.14% increase in total ice sheet volume above floatation over 100 years for a continent wide control simulation using ISSM and Pattyn et al.^69^ reported 0.2–0.3% increase for a continent wide simulation using the f.ETISh model.

## Supplementary information

Supplementary Information

## Data Availability

We use publicly available datasets that are described above in the Methods section on datasets to set up our model geometry and boundary conditions. The model experiment output files from this study can be accessed from the following Zenodo repository: 10.5281/zenodo.6870567.

## Code availability

We use ISSM code version 4.16, which can be downloaded from the ISSM website (https://issm.jpl.nasa.gov/download/). Our script to generate figures can also be found in the Zenodo repository (10.5281/zenodo.6870567).
