# Supplementary material for: Ice mass loss sensitivity to the Antarctic ice sheet basal thermal state
Source: Nat Commun. 2022 Sep 14;13:4957. doi: 10.1038/s41467-022-32632-2 (PMC9474861; doi:10.1038/s41467-022-32632-2)
Supplement: Supplementary file 1 — Supplementary Information [file 41467_2022_32632_MOESM1_ESM.pdf]

Supplementary Information for

Ice mass loss sensitivity to the Antarctic ice sheet basal thermal state

Eliza J. Dawson[1*], Dustin M. Schroeder[1,2], Winnie Chu[3], Elisa Mantelli[4,5], Hélène Seroussi[6]

[1] Department of Geophysics, Stanford University

[2] Department of Electrical Engineering, Stanford University

[3] School of Earth and Atmospheric Science, Georgia Institute of Technology

[4] Institute for Marine and Antarctic Studies, University of Tasmania

[5] The Australian Centre for Excellence in Antarctic Science, University of Tasmania, Hobart, Australia

[6] Thayer School of Engineering, Dartmouth College

*Corresponding author

This file includes:

Supplementary Note 1

Supplementary Note 2

Supplementary Note 3

Supplementary Note 4

Supplementary Note 5

Supplementary References

Supplementary Note 1: Basal thawing experiments to test the robustness of results

In addition to the four experiments discussed in the main text, we run five more experiments to test the robustness of our results to modifications in how we formulate the experiments. We consider the following alterations: (i) an experiment where the model is run with a Budd friction law[1] instead of a Weertman friction law[2], (ii) an experiment where the friction coefficient is modified by reducing it by a percent of its original value to match the mean of the thawed friction coefficient distribution, (iii) an experiment with a larger experiment zone, where the zone is defined as anywhere within 100 km of ice flowing at a rate of at least 50m/yr (vs. 100m/yr in the main text experiments). Our experimental procedure for each of these additional experiments follows the same process as before: we start by defining a basal temperature range below the melting point which we consider as thawable; we identify thawable regions in our modeled initial ice sheet state; in these regions we reduce the basal friction coefficient (α) to simulate thawing; and we then run the model forward for 100 years. All experiments are summarized in Supplementary Table 1.

i. Sensitivity to the choice of friction law

We run Experiments 1 and 4 with a Budd[1] friction law as comparison to our results in the main text that use a and Weertman[2] friction law. The Budd friction law includes effective pressure, which is set at the ice overburden pressure and therefore evolves with the ice thickness. The results from both overall show that the Enderby-Kemp region plus the C'-D' coast spanning George V Land and Wilkes Land experience the highest rates of relative mass loss (Supplementary Fig. 1). However, the specific part of the C'-D' coast that is most responsive does depend on the choice of friction law with Budd simulations

highlighting Wilkes Land (C'-D) and Weertman simulations highlighting George V Land (D-D'). This is also reflected in ice thickness and surface velocity differences for these regions (Supplementary Fig. 1 map). Some glaciers in George V Land flow comparatively slower with a Budd friction law than with a Weertman friction law. In George V Land, there is also more thinning inland in Weertman simulations. Meanwhile in the Wilkes Land basin the differences in velocity and thickness are variable across the outlet glaciers in the region (Supplementary Fig. 1 map).

The differences in mass loss results from Budd and Weertman experiments comes from the added influence of effective pressure in the Budd friction law (Eq. S2). In the Budd simulations, ice flow is responding to both our prescribed alterations to the friction coefficient and to changes in effective pressure. Additionally, our prescribed friction coefficient alterations have a relatively smaller influence on sliding in regions where the effective pressure term is larger. These factors make it harder to distinguish an ice response due to the onset of thaw from an ice response due to changes in effective pressure. In the Budd simulations, when the friction coefficient term is reduced, not only can this facilitate faster sliding but effective pressure can also evolve due to ice thickness changes.

In the George V Land basin, there are patches where effective pressure increases beneath fast flowing outlet glaciers over the 100-yr simulations (Supplementary Fig. 2). This increase in effective pressure makes the bed stickier and results in a velocity slowdown, which is enough to result in a lower rate of mass loss for simulations with Budd friction (up to ~10Gt/yr) than for the corresponding simulations with Weertman friction (up to ~20Gt/yr). In the Wilkes Land Basin, effective pressure increases at the locations of outlet glaciers, most notable being Totten Glacier (Supplementary Fig. 2). Despite the

increase in effective pressure and thickening of these glaciers, this does not outweigh the effect of inland thinning and some isolated spots of thinning along the coast but between glaciers where the velocity increases. As a result, the net signal is more mass loss in Wilkes basin for simulations with Budd friction (up to ~30Gt/yr) than with Weertman friction (up to ~20Gt/yr).

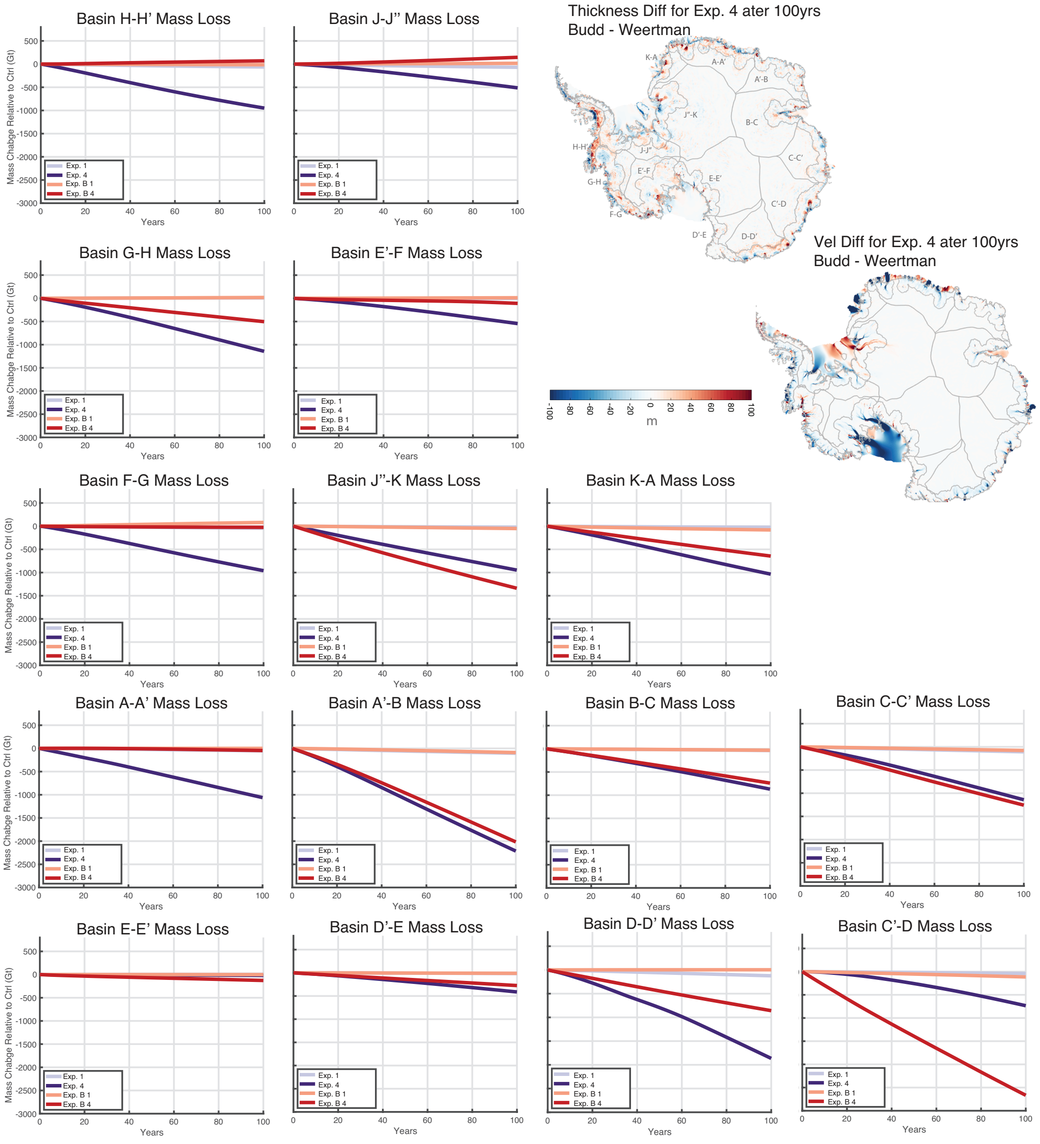

Supplementary Figure 1: Comparisons of experiments run with a Weertman friction law (Exp. 1 and Exp. 4) vs a Budd friction law (Exp. B 1 and Exp. B 4) and the impact it has on the 100-yr mass loss relative to the control. Plots show the changes in mass for each Antarctic drainage basin (defined by E. Rignot and J. Mouginot and used by IMBIE-3[3]) Maps show the difference in ice thickness and surface velocity for Budd vs. Weertman at the end of exp. 4.

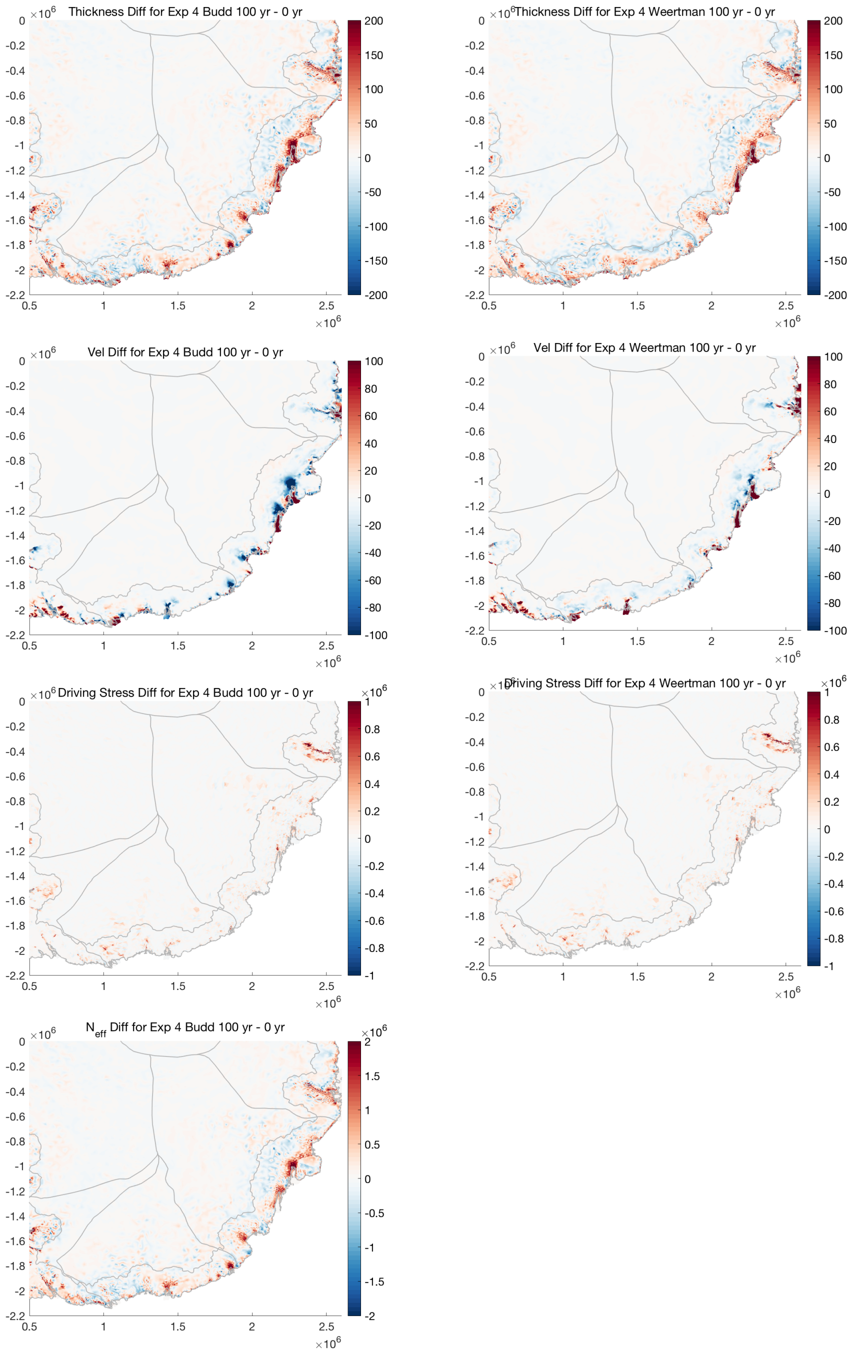

Supplementary Figure 2: Changes in East Antarctica (basins D-D' and C'-D) over 100 years for simulations with Weertman and Budd friction laws. Top row shows thickness changes for Budd and Weertman friction laws, second row shows surface velocity changes, third row shows changes in driving stress and bottom row shows change in effective pressure.

ii. Sensitivity to friction coefficient modification method

As a comparison, we ran the two extreme experiments (Experiments 1 and 4) with a different method to modify α in the thawable regions. This method reduces α by a percentage of its original value, where the reduction percentage corresponded to difference in the means for the α distributions for thawed and thawable regions. Supplementary Fig. 3 shows the results of the percent α modification method (Exp. P 1 and Exp. P 4) compared to the experiments using the CDF α modification method (Exp. 1 and Exp. 4). In all basins, the percent method produces equal or greater mass loss. The East Antarctic coastline from C-D' stands out because the percent method produces at least double the mass loss as the CDF method: in these regions, the ice flow is much higher for the percent method than the CDF method, as seen in the surface velocity map in Supplementary Fig. 3.

The goal behind comparing the percent and CDF α alteration methods was to examine the following tradeoff: The CDF method prioritizes maintaining geographic and geologic continuity by keeping the same shape of the CDF thawed distribution through matching F(x) values to transition from thawable to thawed. Meanwhile, the percent method prioritizes maintaining the structure and spatial patterning of flow by uniformly reducing thawable α values by a percent of their original values. The results show that the method to modify the friction coefficient has virtually no effect on Experiment 1. For Experiment 4, the percent method results in higher mass loss for some regions, especially the higher mass loss regions of East Antarctica, suggesting that the CDF α modification method produces more conservative estimates of mass loss. Since both methods bring important considerations (geologic continuity and the structure of flow) it is important to consider both sets of results in assessments of future mass loss scenarios due to basal thaw.

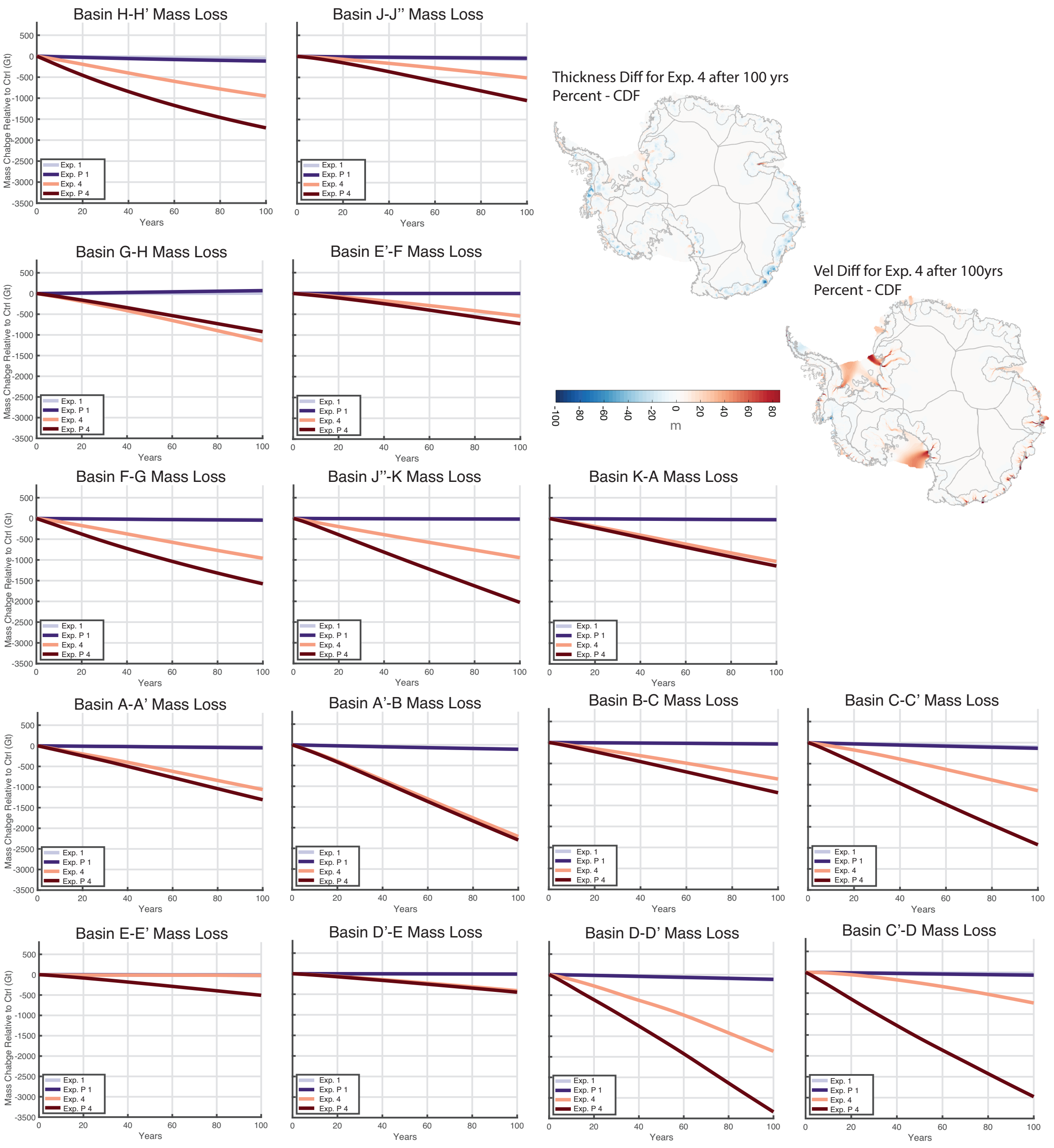

Supplementary Figure 3: Comparison of experiment techniques to modify the friction coefficient and the impact it has on 100-yr mass loss relative to the control run. The simulations with the percent modification method for α are Exp. P 1 and Exp. P 4 and the simulations with the CDF modification method for α are Exp. 1 and Exp. 4. The maps of Antarctica show the differences in thickness and surface velocity that result from the different experiment techniques.

iii. Sensitivity to experiment zone size

As a comparison, we run Experiment 4 with a larger experiment zone size (Exp. Z 4) and compare it to the original zone size (Exp. 4) (Supplementary Fig. 4). The larger zone size is defined as anywhere within 100 km of ice flowing at a rate of at least 50m/yr (vs. 100m/yr in the original experiment). The results show similar mass loss regardless of the difference in experiment zone size definition, which suggests that the results are relatively insensitive to this choice.

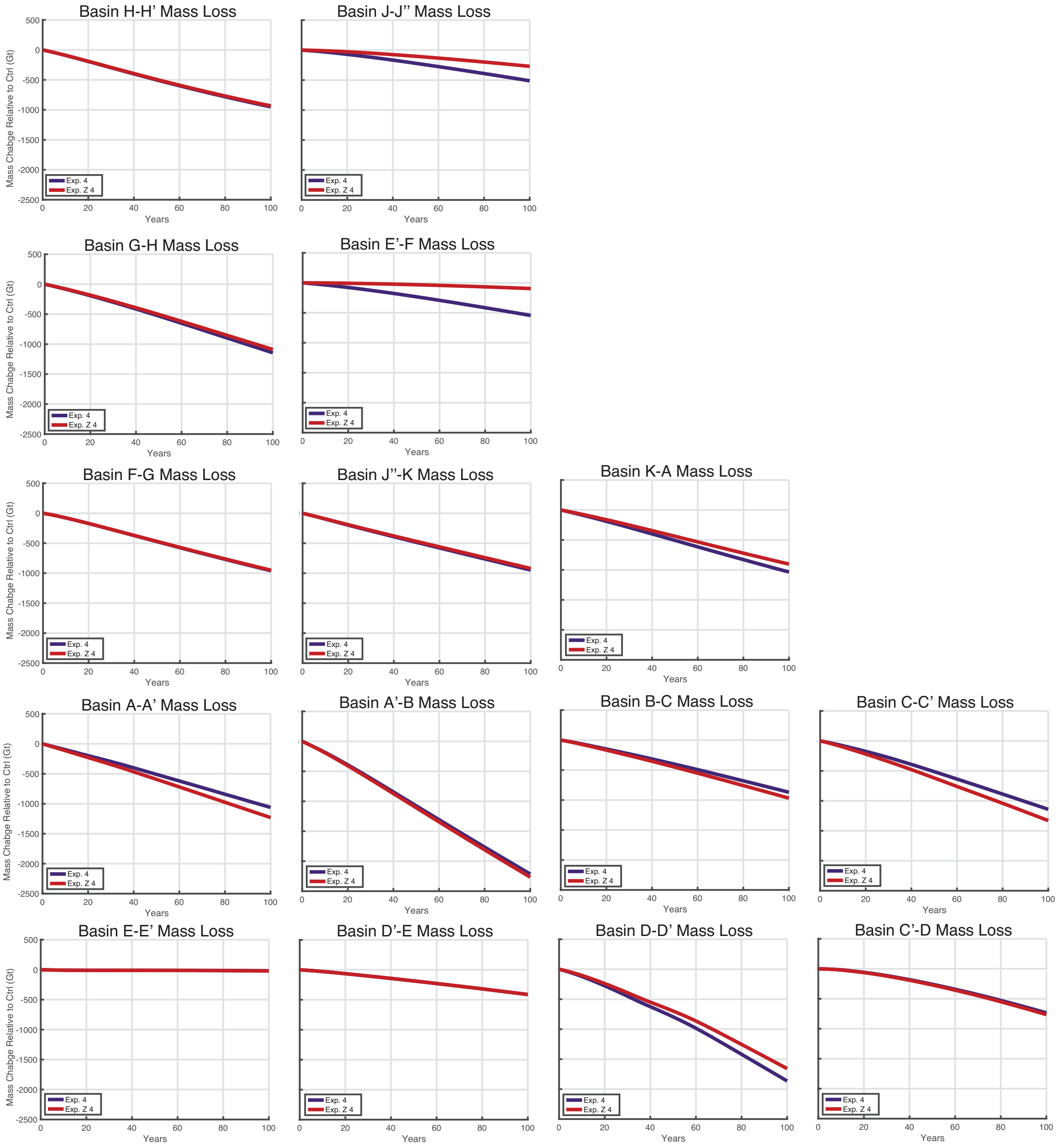

Supplementary Figure 4: Comparisons of Experiment 4 with a different spatial extent of the experiment zone in each drainage basin and the effect that has on mass loss relative to the control. The simulation with the larger zone size is Exp. Z 4 and the simulation with the original zone size Exp. 4.

Thaw Experiment	Frozen	Thawable	Thawed	Friction Law	α Modification Method	Experiment Zone Size
Exp. 1	< -2°	-2° — PMP	At PMP	Weertman	CDF	Original
Exp. 2	< -5°	-5° — PMP	At PMP	Weertman	CDF	Original
Exp. 3	< -8°	-8° — PMP	At PMP	Weertman	CDF	Original
Exp. 4	< -12°	-12° — PMP	At PMP	Weertman	CDF	Original
Control	< PMP	N/A	At PMP	Weertman	CDF	Original
Exp. P 1	< -2°	-2° — PMP	At PMP	Weertman	Percent	Original
Exp. P 4	< -12°	-12° — PMP	At PMP	Weertman	Percent	Original
Exp. Z 4	< -12°	-12° — PMP	At PMP	Weertman	CDF	Larger
Exp. B 1	< -2°	-2° — PMP	At PMP	Budd	CDF	Original
Exp. B 4	< -12°	-12° — PMP	At PMP	Budd	CDF	Original
Control B	< PMP	N/A	At PMP	Budd	CDF	Original

Supplementary Table 1: Summary of all thawing experiments. This includes the basal temperature delineations for frozen, thawed and thawable, the friction law used (Weertman or Budd, see Note 1 (i), the α alteration method (CDF or Percent, see Note 1 (ii), and experiment zone size (original or larger, see Note 1 (iii). Control simulations with no prescribed thawing serve as a point of comparison for experiments with a Weertman friction law as well as a Budd friction law.

Antarctic Region	Region Area (km^2)	Exp. Zone Area (km^2)	Thawable Area	α to α_{exp} Change	Relative Mass Loss
**Siple Coast (E'-F)**	8.0 x 10^5	4.7 x 10^5	< 43%	Moderate	Low
**Marie Byrd Land (F-G)**	1.3 x 10^5	1.3 x 10^5	< 42%	Moderate	Moderate
**Amundsen (G-H)**	4.2 x 10^5	2.8 x 10^5	< 53%	Low	Moderate
**Bellingshausen (H-H')**	0.7 x 10^5	0.7 x 10^5	< 53%	Moderate	Moderate
**Ellsworth (J-J'')**	6.2 x 10^5	3.8 x 10^5	< 32%	Moderate	Low
**Shackleton-Pensacola (J''-K)**	20.5 x 10^5	3.8 x 10^5	<27%	High	Moderate
**Dronning Maud Land (K-A)**	2.6 x 10^5	1.4 x 10^5	<48%	Moderate	Moderate
**Dronning Maud Land (A-A')**	7.7 x 10^5	3.0 x 10^5	<41%	Moderate	Moderate
**Enderby Land (A'-B)**	6.5 x 10^5	3.0 x 10^5	<47%	Moderate	High
**MacRobertson Land (B-C)**	13.1 x 10^5	2.5 x 10^5	<36%	Moderate	Moderate
**Princess Elizabeth Land (C-C')**	7.1 x 10^5	2.6 x 10^5	< 54%	Moderate	Moderate
**Wilkes Land (C'-D)**	11.6 x 10^5	2.8 x 10^5	<45%	Moderate	Moderate
**George V Land (D-D')**	7.0 x 10^5	1.7 x 10^5	< 58%	High	High
**Transantarctic Mountains (D'-E)**	4.2 x 10^5	2.2 x 10^5	<18%	High	Low
**Transantarctic Mountains (E-E')**	15.8 x 10^5	2.7 x 10^5	<18%	High	Low

Supplementary Table 2: Synthesis of the regional mass loss and contributing factors from the basal thawing experiments. We classify the change in α going from thawable to thawed as low if the values are less than 30 $(Pa/yr\ m)^{1/2}$ different, moderate if the values are less than 50 $(Pa/yr\ m)^{1/2}$ different, and high if the values are greater than 50 $(Pa/yr\ m)^{1/2}$ different. The regional mass loss is calculated from the change in ice volume above flotation for each drainage basin (experiment – control). In order to quantify the mass loss response for each basin, we categorize low as less than 500 Gt of mass loss for the most extreme thawing experiment, moderate between 500 and 1500 Gt of mass loss for the most extreme thawing experiment, and high as more than 1500 Gt of mass loss.

Supplementary Note 2: CDF distributions of the basal friction coefficient (α) for all drainage basins

We calculate the CDF distributions for thawed, thawable, and frozen bed regions of the experiment zone in each drainage basin. The results for Experiment 4 are plotted in Supplementary Fig. 5. The qualitative shape of the CDF distributions is fairly insensitive to how the definition of thawable changes for each of the experiments. As an example, Supplementary Fig. 6 shows the CDFs for region D-D' for Experiments 1-4 where it is apparent that the distributions of α for thawed, thawable, and frozen are independent of how the definition of thawable varies across Experiments 1-4.

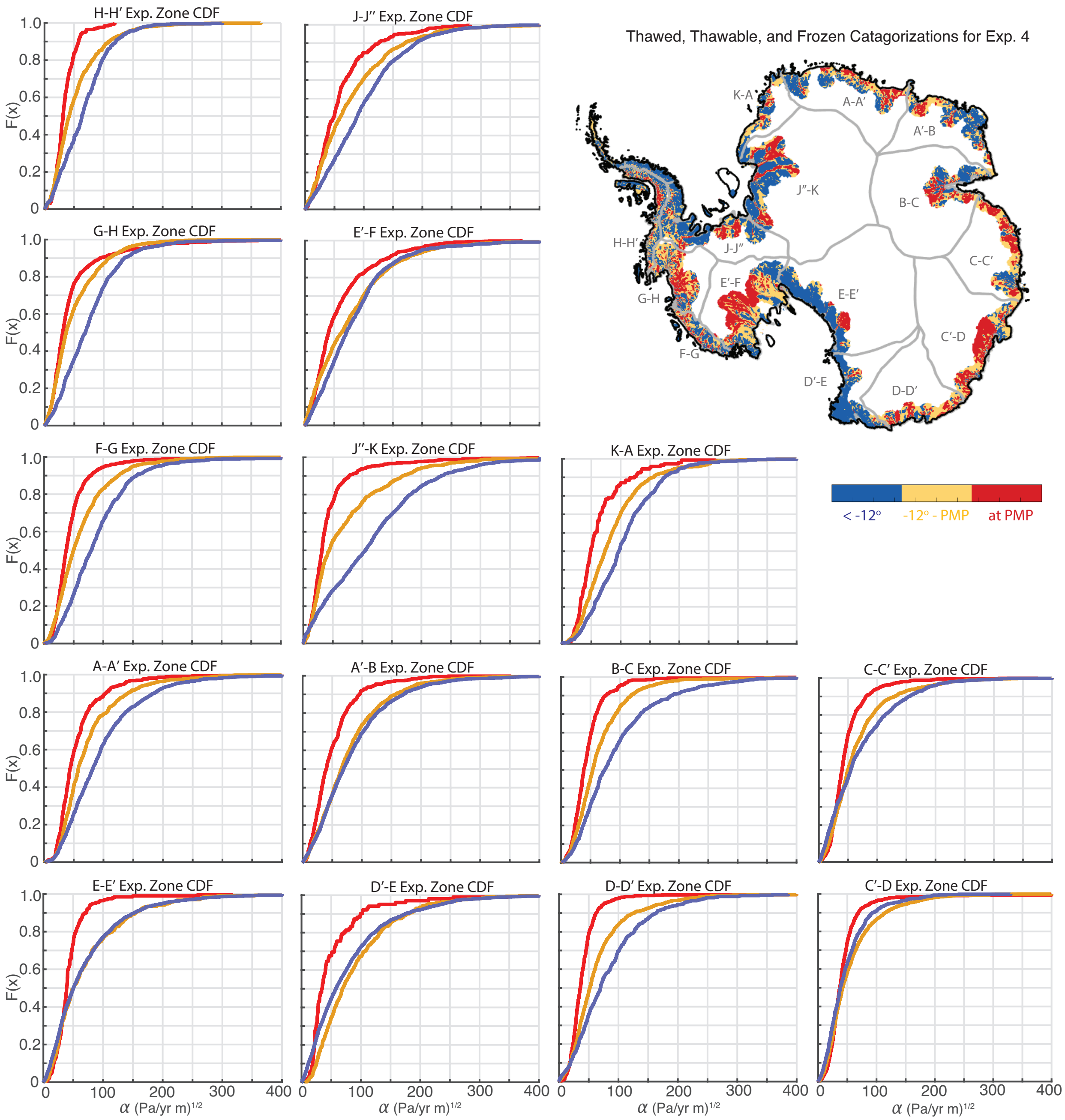

Supplementary Figure 5: Basal friction coefficients (α) for frozen, thawed, and thawable bed regimes from Experiment 4. Each plot shows the empirical cumulative density function (CDF) for α in the thawed (red), thawable (yellow), and frozen (blue) regions for each drainage basin experiment zone. The top right map shows the spatial extent of the thermal regimes for Experiment 4.

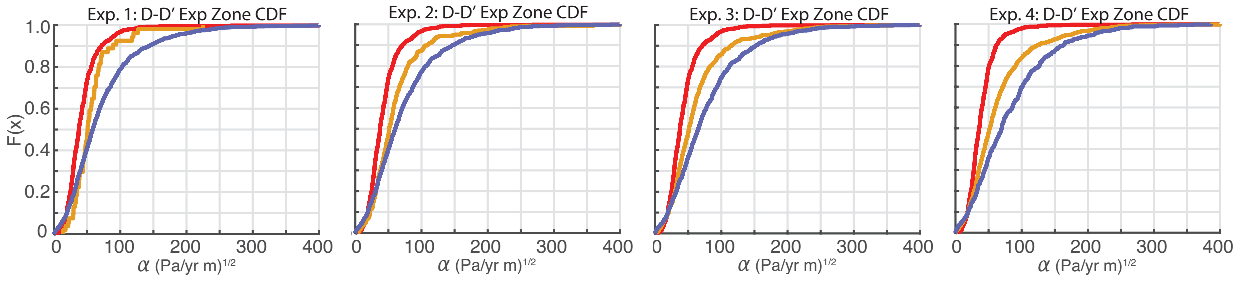

Supplementary Figure 6: Comparisons of CDF distributions for Experiments 1-4 for region D-D'. Similar results are found across all drainage basins.

Supplementary Note 3: Changes in ice sheet thickness for Experiments 1-4

To further investigate our mass loss results, we also examine the changes in ice thickness at the end of each 100-year experiment (experiment – control), as shown in Supplementary Fig. 7. There is virtually no change in thickness for Experiment 1 compared to the control. In contrast, for the more extensive thawing experiments, thickening can be seen along the coast - especially for fast flowing glaciers and ice streams - and thinning interior of that. This pattern is strongest in the basins with the highest mass loss. While this pattern is an interesting result, investigating how thickness changes could feedback on basal thermal state changes is beyond the scope of this study.

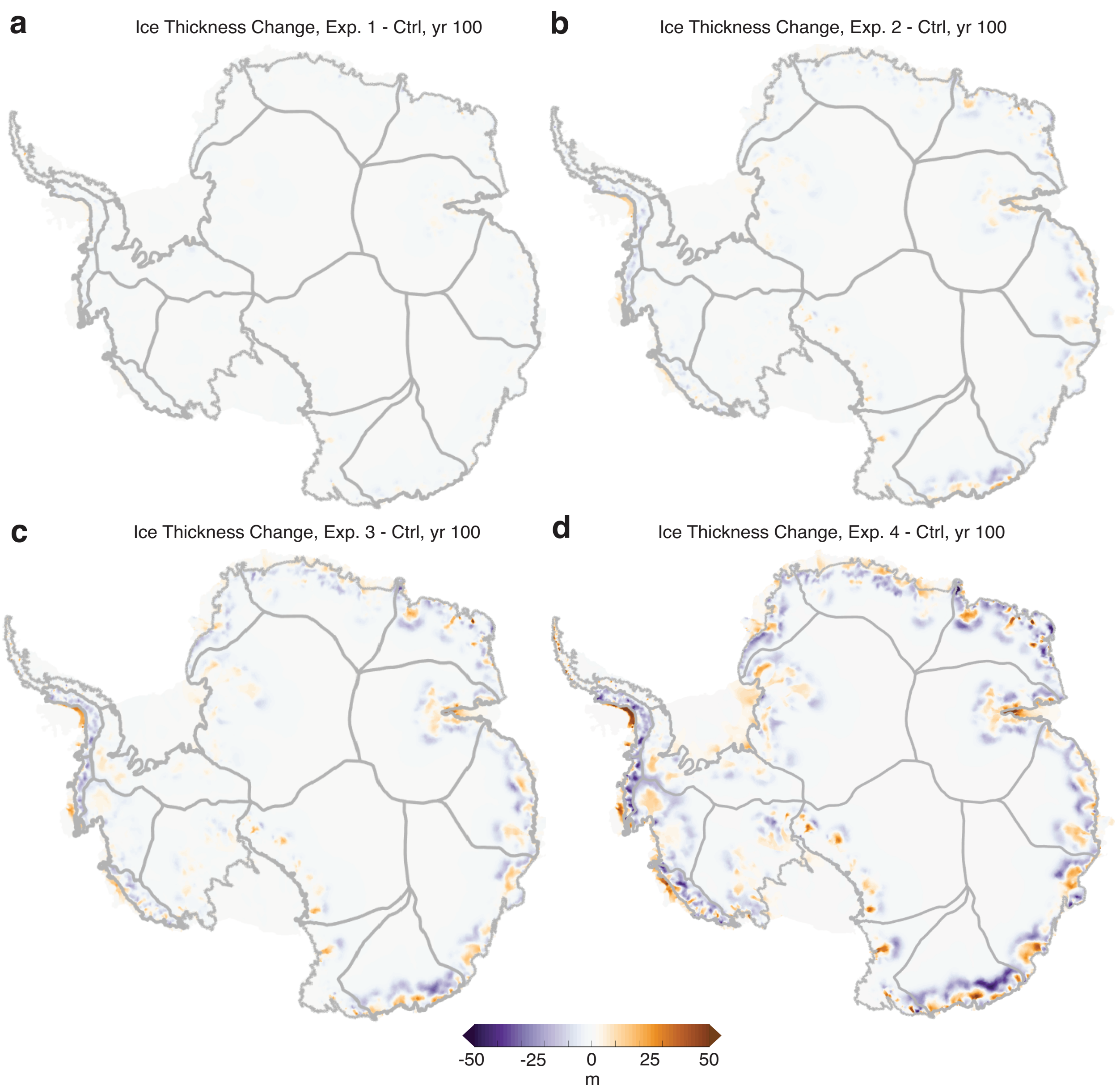

Supplementary Figure 7: a) Change in ice thickness after 100 years for Experiment. 1 relative to the control run. b-d) Same as a) but for Experiments 2-4.

Supplementary Note 4: Grounded ice basal melt rate change over time

Over the course of the 100-year simulations, the modeled basal melt rates in the thawable zones are positive (Supplementary Fig. 8). This suggests that the thawable bed regions that undergo thawing in the experiments would not refreeze. This is an important consideration since the experiments follow a predefined alteration in α corresponding to thawing that is not allowed to further adjust. Ultimately, however, a continent-scale ice sheet model with temperature-dependent sliding physics is needed to examine the self-reinforcing feedbacks that could either lead to the ice thinning and eventually refreezing to the bed, or permit sustained thawed conditions if frictional heating is great enough[4,5].

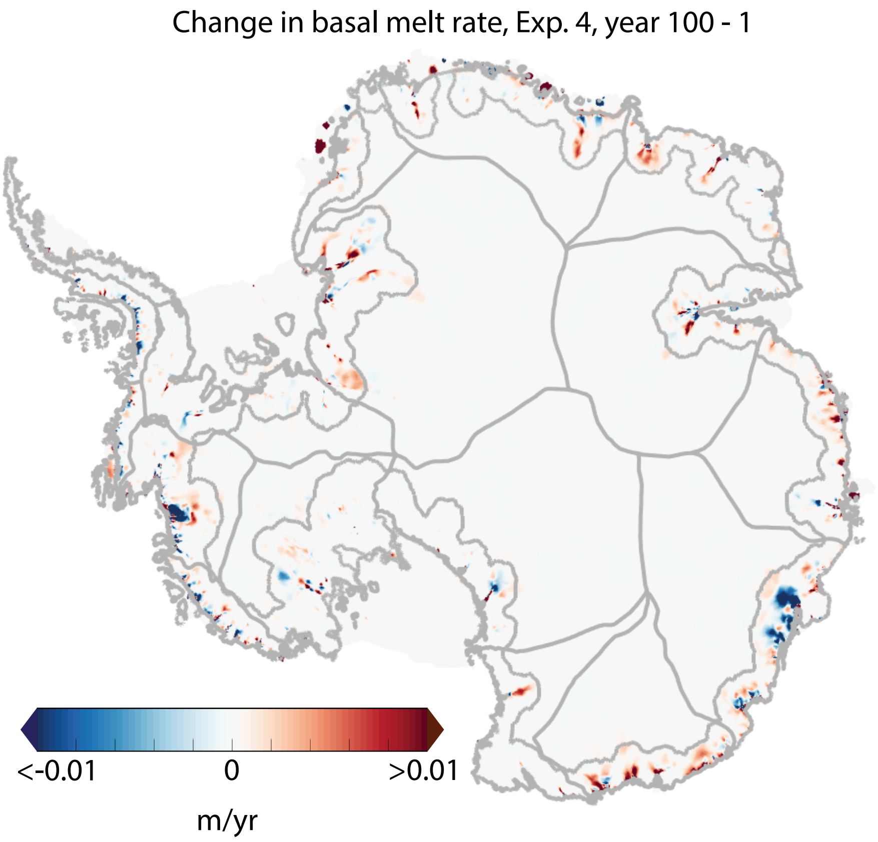

Supplementary Figure 8: Change in modeled basal melt rates at the end of Experiment 4 relative to the start of the simulation.

Supplementary Note 5: Figures from ISSM initialization

Our ISSM initialization is discussed in the Methods section of the main manuscript. The figures we discuss in that section are attached below.

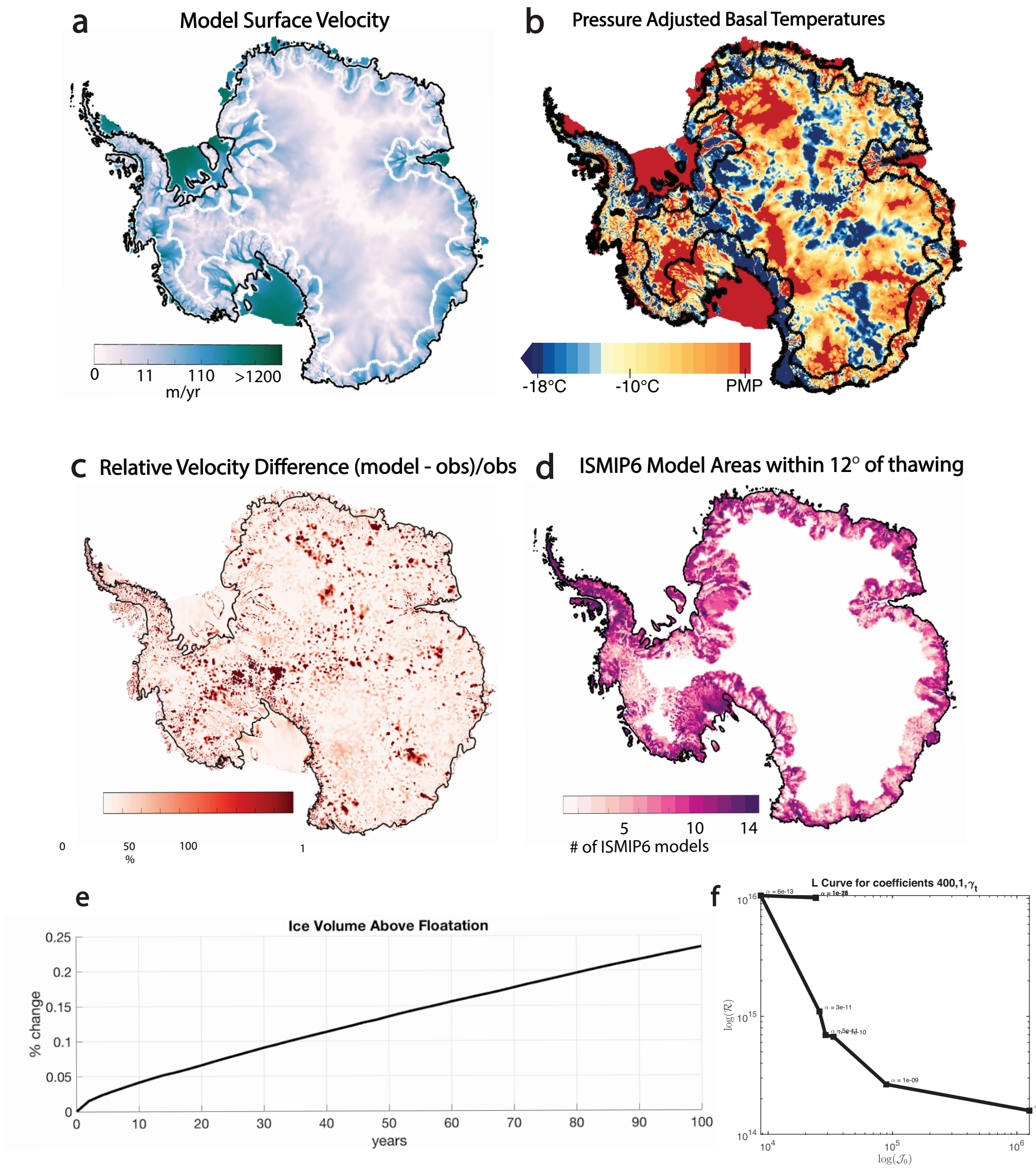

Supplementary Figure 9: a) Modeled Surface Velocity. b) Modeled basal temperatures adjusted for the pressure melting point. c) Relative difference in modeled vs observed surface velocity. d) Number of ISMIP6 models in the thawable range within our experiment zone. White line in a) and black line in b) outline the interior extent of the experiment zones. e) Change in ice volume above flotation over 100 years for the control simulation, where α is unperturbed from its initial inferred values. f) Results from L curve analysis following procedure from Morlighem et al., 2013[6]

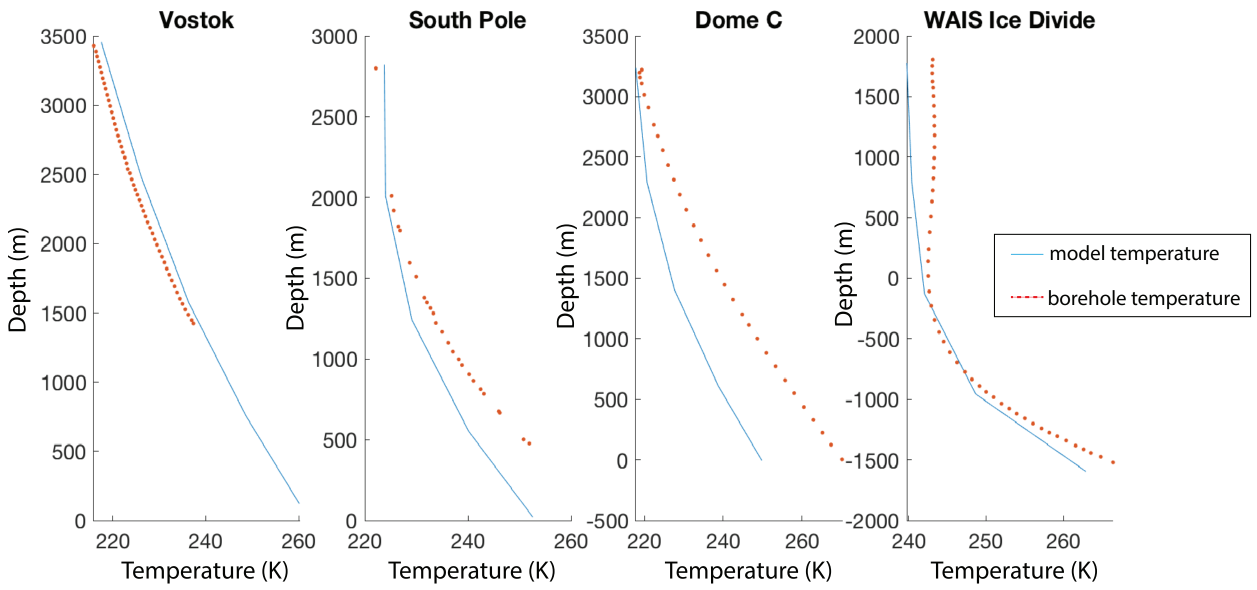

Supplementary Figure 10: Temperature comparison between modeled temperature field (blue line) and borehole observations (red dots) from Lake Vostok, South Pole, Dome C, and WAIS Ice Divide[7–10].

Supplementary References

1. Budd, W., Jenssen, D. & Smith, I. A Three-Dimensional Time-Dependent Model of the West Antarctic Ice Sheet. *Ann. Glaciol.* **5**, 29–36 (1984).
2. Weertman, J. On the Sliding of Glaciers. *J. Glaciol.* **3**, 33–38 (1957).
3. Rignot, E. *et al.* Four decades of Antarctic Ice Sheet mass balance from 1979–2017. *Proc. Natl. Acad. Sci.* **116**, 1095–1103 (2019).
4. Mantelli, E. & Schoof, C. Ice sheet flow with thermally activated sliding. Part 2: the stability of subtemperate regions. *Proc. R. Soc. Math. Phys. Eng. Sci.* **475**, 20190411 (2019).
5. Schoof, C. & Mantelli, E. The role of sliding in ice stream formation. *Proc. R. Soc. Math. Phys. Eng. Sci.* **477**, rspa.2020.0870, 20200870 (2021).
6. Morlighem, M., Seroussi, H., Larour, E. & Rignot, E. Inversion of basal friction in Antarctica using exact and incomplete adjoints of a higher-order model. *J. Geophys. Res. Earth Surf.* **118**, 1746–1753 (2013).
7. Salamatin, A. N., Lipenkov, V. Y. & Blinov, K. V. Vostok (Antarctica) climate record time-scale deduced from the analysis of a borehole-temperature profile. *Ann. Glaciol.* **20**, 207–214 (1994).
8. Price, P. B. *et al.* Temperature profile for glacial ice at the South Pole: Implications for life in a nearby subglacial lake. *Proc. Natl. Acad. Sci.* **99**, 7844–7847 (2002).
9. Clarke, A. *et al.* A Low Temperature Limit for Life on Earth. *PLOS ONE* **8**, e66207 (2013).
10. Cuffey, K. M. *et al.* Deglacial temperature history of West Antarctica. *Proc. Natl. Acad. Sci.* **113**, 14249–14254 (2016).
